# CRISPR‐based reagents to study the influence of the epigenome on gene expression

**DOI:** 10.1111/cei.13190

**Published:** 2018-09-11

**Authors:** P. Lavender, A. Kelly, E. Hendy, P. McErlean

**Affiliations:** ^1^ Peter Gorer Department of Immunobiology, School of Immunology and Microbial Science MRC and Asthma UK Centre in Allergic Mechanisms of Asthma, King’s College London London UK

**Keywords:** gene regulation, molecular biology, transcription factors

## Abstract

The use of epigenome editing is set to expand our knowledge of how epigenetic landscapes facilitate gene expression capacity within a given cell. As epigenetic landscape profiling in health and disease becomes more commonplace, so does the requirement to assess the functional impact that particular regulatory domains and DNA methylation profiles have upon gene expression capacity. That functional assessment is particularly pertinent when analysing epigenomes in disease states where the reversible nature of histone and DNA modification might yield plausible therapeutic targets. In this review we discuss first the nature of the epigenetic landscape, secondly the types of factors that deposit and erase the various modifications, consider how modifications transduce their signals, and lastly address current tools for experimental epigenome editing with particular emphasis on the immune system.

## Epigenetics

The completion of the draft sequence of the human genome in 2001 1 heralded intense interest in the management and implementation of gene expression programmes, understanding the basis of cell differentiation and cell type specification. These studies have begun to reveal how an individual cell type establishes and maintains its ability to express a distinct repertoire of genes and how this output capacity can be modulated under the influence of intrinsic and extrinsic stimuli. Consortium projects such as Roadmap 2 and Encyclopedia of DNA Elements (ENCODE) 3 have begun the job of identifying all functional elements within the human genome. This task encompasses mapping RNA transcripts in all cell types, mapping the location of DNA associating with modified histones and transcription factors and mapping modification of DNA itself by methylation. In an ENCODE update in 2012 3, with comprehensive analysis of fewer than 20% of the known transcription factors encoded by the human genome, of half the DNA and histone modifications and potentially a similar percentage of known cell types, the data were startling. The studies have led to fundamental insights into genome organization and utility, as exemplified by findings that greater than 60% of bases in the genome are represented in long RNA molecules yet only one‐tenth of these are constituents of the roughly 20 000 known genes in the genome, that there are greater than 400 000 regions of the genome that have ‘enhancer‐like’ chromatin features and that there are more than 70 000 regions with ‘promoter‐like’ features. Even with these incomplete projects, it is clear that there are significant gaps in our understanding of the labyrinthine influence of regulatory RNAs, transcription factors and chromatin states that contribute to gene expression pathways.

Epigenetics research is complicated by a number of factors: first, because epigenomes are cell type‐specific, a challenge has been to generate purified cell types in sufficient numbers to exploit technologies that can report on the landscape in an unbiased manner. Cell types that arise during early differentiation represent a particular challenge for isolation for both numerical and ethical reasons. In adults, while cell purification is relatively straightforward in mature immune systems, emerging information from single‐cell sequencing has begun to reveal an expanded repertoire of distinct cell types, as exemplified with subtypes of dendritic cells 4]. The consequence of this information is that the spectrum of cell types on which epigenetic landscapes and their associated transcriptomes might be analysed is greater than thought previously. Clear inroads are being made in this area through the Human Cell Atlas project 5, but even in these situations different transcriptomes may be influenced by different activation status of cells within a population. Tissue resident immune cells present particular problems; these cells, such as T resident memory cells, may lack substantial presence in the bloodstream, as demonstrated conclusively by parabiosis experiments 6. The challenge is therefore how to collect sufficient of these cells in order to establish their epigenetic footprint, particularly in human disease scenarios.

Certain immune cells are often considered to be phenotypically plastic 4, and this property is important in the rapid adaptation to external stimuli. Functional plasticity, however, brings into question whether the gene expression patterns used to define individual cell types are sufficiently invariant to discriminate stable populations. The use of t‐Distributed Stochastic Neighbour Embedding (tSNE) 7 analysis is making identification of cells having similar phenotypical characteristics more amenable and permits identification of expression signatures that may allow subsequent purification by flow cytometry.

A further challenge is technical, and derives from the fact that sequencing‐driven techniques such as ChIPseq and ATACseq enable population‐level analysis which need careful interpretation in order to determine for how many cells in that population the landscapes derived are accurately representative. Epigenome‐wide analysis from single cells and small numbers of cells is now the subject of numerous applications 8, 9, 10, and data derived from single‐cell epigenome analysis will no doubt be of great importance in understanding the mechanism of cell differentiation.

## Chromatin organization

**Figure 1:**
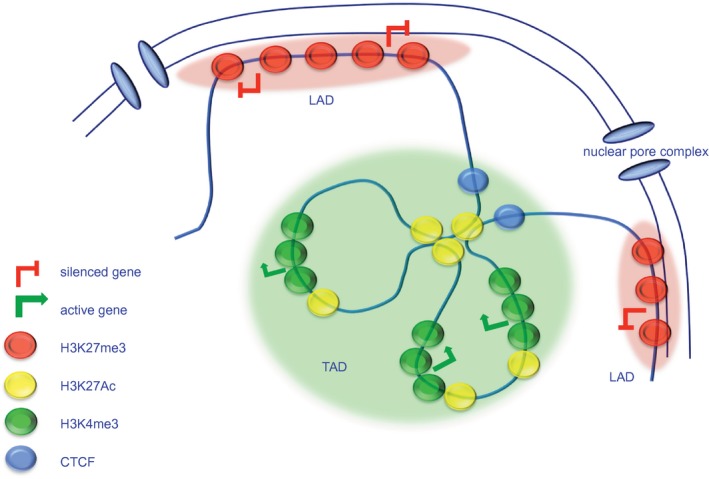
Chromatin domains. Chromatin is organized into lamina‐associated domains (LADs) and topologically associating domains (TADs). LADs are enriched in genes that are either not expressed or are expressed at low levels. DNA sequences within individual TADs interact with one another physically with a greater frequency than they do with sequences outside the TAD suggesting that regulatory domains influencing expression of an individual gene will be constrained to a particular TAD.

The ability to control transcriptional output of any cell is impacted at a number of levels. Experiments on higher‐order chromatin have started to reveal the complex communications that exist between chromosome domains and the establishment of chromosome territories within the nucleus 11. Chromosome conformation analysis has determined the presence of topologically associated domains (TADs) and lamina‐associated domains (LADs) 12. Genes within TADs are more likely to be regulated by domains within the same TAD, and at the borders of these lie boundary elements that separate individual TADs from one another. LADs are regions of chromatin that associate with the nuclear lamina and tend to be enriched with repressed genes (Fig. 1). Within these distinct chromatin regions, gene expression is impacted by the immediate epigenetic landscape. ENCODE 3 and Roadmap 2 have worked to discover what combination of epigenetic marks are found at individual genes, and what transcription factors and co‐factors assemble to deposit, maintain and modify these marks. While ENCODE sought to catalogue the regulatory elements of human cells grown in culture, Roadmap epigenomics expanded this repertoire by studying cells derived directly from human tissues in health and disease.

The two primary determinants of epigenetic landscapes remain the histone code and DNA methylation. Consequently, the substrates are the histone proteins which constitute the protein component of the nucleosome and, predominantly, the cytosine : guanosine dinucleotide (CpG) of DNA. In this review, we refer only to the canonical targets, histones H2A, H2B, H3 and H4 and modifications thereof, but there is a large body of work studying replacement histones such as H2AZ and H3.3, let alone the linker histone H1, along with non‐canonical targets for DNA methylation.

Understanding what effect specific post‐translational modification of histone proteins has upon gene expression capacity remains an active area of research 13. For the illustrative purposes of this review a compendium of the modifications that assemble around an individual gene and the functions they bestow is not possible. However, consideration of the combinatorial impact of histone methylation, acetylation, phosphorylation, ubiquitylation, crotonylation and a number of other modifications lies at the heart of our ability to understand the impact upon gene expression.

Despite these challenges, expressed genes or genes for which expression is permissive upon receipt of appropriate stimuli within the cell tend to be marked epigenetically in a broadly similar way. Epigenetic landscapes can reveal capacity for both current and predictive future gene transcription compared to RNA signals, which are a record of either current or historical expression which remains in the cell until those RNAs are degraded. An average expressed gene would tend to display a complex pattern of histone modifications which includes but is far from limited to including trimethylation of lysine 4 on histone H3 at the nucleosomes that pack approximately 3 kb of DNA around the transcriptional start site (TSS) 14. Histones around TSSs tend to be acetylated at H3K27 15, while those that demarcate transcriptional regulatory domains such as enhancers may be decorated by both H3K27acetylation and H3K4 monomethylation 16, 17, and these distal regulatory domains may be in anything from within kilobases to megabases away from the genes whose expression they regulate. H3K36 trimethylation is enrichment at transcribed exons 18 and histone acetylation at H3K9, 12 and 14 is also found around expressed genes 19. Expressed genes also display H4K20, H3K27 and H3K9 monomethylation 14 and H3K79 dimethylation 3. A summary of the potential roles of some of these modifications is shown in Table 1.

**Table 1:** Location and characteristics of histone modifications cited in the text (adapted from reference 3)

Histone modification	Signal characteristic	Proposed function
H3K4me1	Peak/region	Marks regulatory domains, also enriched downstream of transcriptional starts
H3K4me2	Peak	Marks regulatory elements at transcription starts
H3K4me3	Peak	Marks regulatory domains preference for active promoters
H3K9Ac	Peak	Marks regulatory domains
H3K9me1	Region	Marks actively transcribed genes, preference for 5' ends
H3K9me3	Peak/region	Repressive mark
H3K27Ac	Peak	Mark of regulatory domains, distinguishes active enhancers and promoters
H3K27me1	Region	Marks active promoters
H3K27me3	Region	Repressive marks found at silenced loci deposited by PRC2
H3K36me3	Region	Elongation mark associated with transcribed exons
H3K79me2	Region	Transcription‐associated mark
H3K20me1	Region	Marks 5' end of active genes
H2AK119Ub	Region	Repressive mark found at silenced loci deposited by PRC1

PRC = polycomb repressive complex.

In terms of genes that are epigenetically silenced, histones associated with the transcribed region and 3' and 5' intergenic regions of DNA are often decorated by H3K27 and H3K9 trimethylation 14 and H2AK119 ubiquitylation 20. Thus, it becomes apparent that depending upon the nature of the modification, specific amino acids such as K9 and K27 on histone H3 can convey signals of both permissibility and prevention of transcription. How this antagonism of regulatory complexes is regulated underpins gene expression potential. How these modifications are assembled in a temporal sense is not clear, nor is it clear how precise recruitment of the dizzying numbers of catalytic activities that shape the landscape is enabled. Abundant evidence demonstrates the fact that numerous epigenome regulators exist within multi‐protein complexes such as polycomb, trithorax and Spt‐Ada‐Gcn5 acetyltransferase (SAGA) which regulate silencing, expression capacity and nucleosome remodelling, to name but three 21, 22, 23.

Cytosine methylation is the most prevalent modification of DNA. In canonical DNA methylation, methyl groups are added to position 5 of the pyramidine ring of deoxycytosine within the context of a CpG dinucleotide. The methylation status of CpG dinucleotides is not uniform among individual genes; rather, CpGs show position‐specific variation in methylation. While the DNA regions proximal to TSSs of genes that are permissive for transcription tend to be depleted of DNA methylation, the areas flanking the TSS may display a greater degree of variation in methylation 24. At genes displaying a greater concentration of CpG dinucleotides around their TSSs (CpG islands) the tendency is for CpG dinucleotides at the flanks (or shores and shelves) of the islands to show most variation in methylation status in distinct cell populations. Genes that are epigenetically silenced may have promoters that are either enriched for methylated cytosines at TSSs or at CpG island shores.

It is likely is that these two gross transcriptional states, being epigenetically silenced or actively transcribed, represent the extremities of the possible gene expression potentials, and an individual gene in any given cell is unlikely to be able to switch easily and rapidly between the two. Variation of transcriptional activity may reflect different regulatory domains being revealed or not revealed in distinct cell states. Alternatively, or additionally, transient expression of key transcription factors such as Jun, Fos and nuclear factor kappa B (NF‐kB) might act upon and modify further a chromatin landscape that is already broadly permissive for transcription.

## Writers and erasers: protein motifs that catalyse deposition and removal of post‐translation histone modifications

One of the key advances to understanding the causal role of epigenetic landscapes on gene expression and cellular phenotype has been the identification of proteins that deposit, remove or signal transducers of histone and DNA modifications, the so‐called writers, erasers and readers of the epigenetic codes.

## Histone methylation

The identification of the SET (Su(var)3‐9, Enhancer‐of‐zeste and Trithorax) domain allowed the enzymes that methylate distinct lysine residues in histones to be characterized 25. Histone methyltransferases now include proteins that are able to mono‐, di‐ or trimethylate both lysine and arginine residues and non‐SET‐containing methyl transferases have also been reported such as the arginine‐targeting protein arginine methyltransferases (PRMT) family 26. These proteins use s‐Adenosyl methionine as a co‐enzyme and methyl donor.

In contrast, Lsd1 (KDM1A), a flavin‐containing amino oxidase, was the first enzyme identified that had the ability to demethylate mono‐ and dimethylated histone H3 lysine 4 histone substrates 27. Subsequently, a second, larger family of Fe (II) and 2‐oxoglutarate oxygenases, which contain a catalytic domain which has been termed the ‘Jumonji domain’, was identified 28. Like the SET domain, the Jumonji domain was found to be present in a large number of histone demethylase proteins, with individual proteins having the ability to attack specific residues of the histone proteins.

## Histone acetylation and crotonylation (HAT)

Both histone acetyl transferase and deacetylase families are characterized by their sequence homologies. There are upwards of 30 distinct proteins in humans with histone acetyl transferase activity, a catalytic activity that transfers an acetyl group from acetyl coenzyme A (CoA) to the ε‐amino group of a histone lysine residue. Some of these proteins can also use crotonyl CoA as their substrate. These HATs are categorized into families, first by their cellular distribution, which comprises either nuclear or cytoplasmic HATs. The nuclear HATs are categorized further by structural homology into three main groups, the GNAT (GCN5‐related N‐acetyltransferases) 29, MYST (MOZ: monocytic leukaemia zinc finger protein), Ybf2/Sas3, Sas2 and Tip60 (Tat interacting protein)^30^ and CBP/p300 families 31.

Four classes of histone deacetylase (HDAC) proteins have been identified, and these classes are again demarcated based upon sequence homologue. Class I HDACs tend to be expressed ubiquitously and compartmentalized to the nucleus 32, whereas class IIA/IIB and 4 shows some cell type restriction; these proteins have a zinc‐dependent catalytic domain 33 (class III HDACs are the sirtuins, which are NAD^+^‐dependent 34.

## DNA methylation and demethylation

The three mammalian DNA methyl transferases (DNMTs), DNMT1, 3a and 3b, catalyse *de novo* and maintenance DNA methylation. DNMT1 is the key maintenance methyl transferase utilizing hemimethylated DNA as its substrate, but can also undertake *de‐novo* methylation. DNMT3A and B are both *de‐novo* methyltransferases but have a lower activity than DNMT1 35.

The ten‐eleven translocation methylcytosine dioxygenase (TET) family initiate a chain of reactions that can ultimately demethylate 5 methylcytosine 36, 37. This process generates a number of intermediates (5‐hydroxy methylcytosine, 5‐formylcytosine and carboxylcytosine) that may also have roles as epigenetic marks 38.

Many of the writers and erasers of histone and DNA modifications have additional motifs outside of their catalytic domains which are responsible for the targeting of the protein to chromatin. These domains include chromodomains which recognize methylated histones, bromodomains which recognize acetylated histone, plant homeodomain (PHD) fingers, TUDOR and WD40 domains (reviewed in 39 **), **demonstrating that both reader and writer functionality can reside within the same protein. In addition, histone and DNA‐modifying proteins assemble into multi‐protein complexes such as Complex of Proteins Associated with Set1 (COMPASS), SAGA and polycomb. In terms of DNA methylation, both methylated and unmethylated CpG dinucleotides are recognized by specific binding proteins. MBD (methyl‐binding domain‐containing) and methyl CpG‐binding proteins (MECP) recognize methylated DNA 40, while unmethylated CpG at TSSs are recognized by the H3K36 demethylase KDM2A 41, providing further evidence for the interplay between histone post‐translational modifications and DNA methylation. The bromodomain‐containing protein BRD4, which is found as a translocation product with the nuclear protein in testis (NUT) protein in human squamous carcinoma, has been targeted using bromodomain inhibitors 42. These compounds block the binding of BRD4 to acetylated histone 4, and a therapeutic strategy using bromodomain inhibitors has been used for mixed‐lineage leukaemia (MLL)‐fusion leukaemia 43.

Cataloguing the epigenetic landscape in health and disease highlights regions of chromatin at which differences occur, but there needs to be a mechanistic correlate or analysis of cause or consequence to determine how these modifications influence gene expression.

Collectively, the identification of the writers and erasers of the epigenetic landscape has presented an opportunity to begin to interrogate the precise influence of epigenetic landscapes upon cellular phenotype and gene expression potential. However, with such a large number of enzymes catalysing post‐transcriptional modification of histones in a tightly controlled temporal manner histone, a key challenge to being able to alter the epigenetic landscape at will has been how to deliver precisely the right catalytic domains of histone or DNA‐modifying enzymes at the right time.

**Figure 2:**
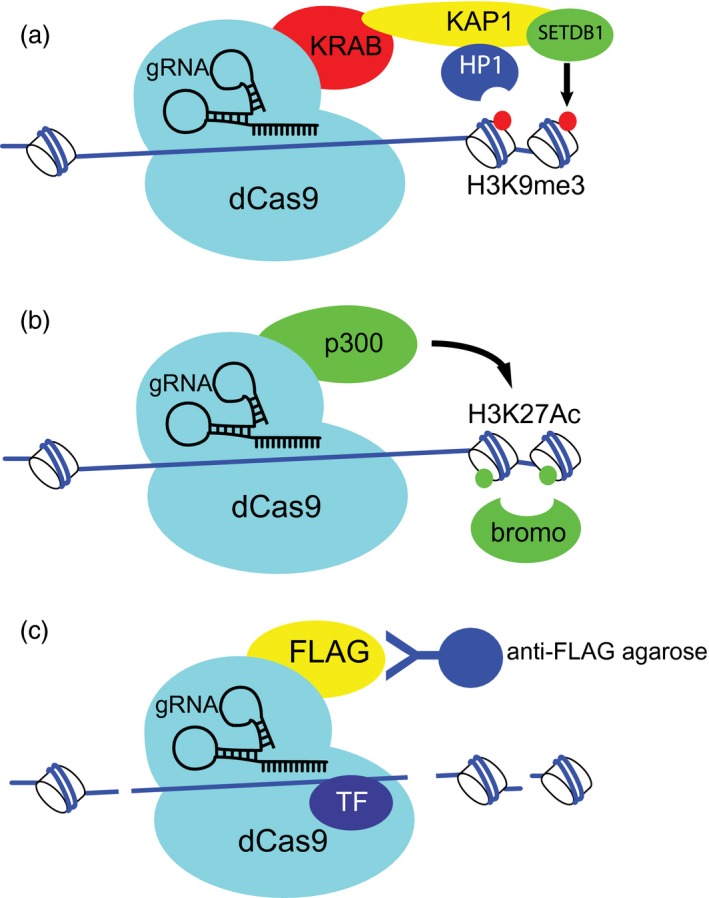
Use of dCas9 fusion proteins in epigenetics. (a) dCas9 is cloned in frame with a Kruppel‐associated box (KRAB) domain which, upon delivery to cells along with specific gRNAs, can mediate gene silencing. Silencing is facilitated by the recruitment of KRAB‐associated protein‐1 (Kap1) to KRAB. Kap1 contains a heterochromatin protein1 (HP1) binding domain which is required for transcriptional repression, and a carboxy‐terminal tandem plant homeodomain (PHD) and bromodomain which can recruit the H3K9 methyl transferase SETDB1 to implement H3K9 methylation. (b) dCas9 is cloned in frame with the catalytic domain of the histone acetyl transferase p300. The histone acetyl transferase (HAT) is able to mediate the acetylation of H3K27 in a gRNA‐dependent manner. H3K27acetylation can be recognized by bromodomain‐containing proteins. (c) dCas9 is cloned in frame with an epitope such as FLAG, which can be used with specific gRNAs to immunoprecipitate fragmented chromatin and proteins bound to it.

This has led to a number of approaches. In the first, the precise nature of the catalytic enzymes is disregarded and the goal is merely to deliver epigenetic marks that are either permissive or refractory for gene expression. Platforms such as zinc finger nucleases 44 and transcription activator‐like effector nucleases (TALENs) 45 were the first reagents to employ these technologies enabling delivery to precise genomic locations. Their use has been eclipsed by the clustered, regularly interspaced, short palindromic repeats (CRISPR)‐Cas9 system 46. In a modification of this system, a nuclease dead version of Cas9 is cloned in frame with transcriptional activators such as VP16, the herpes simplex virus protein vmw65, to deliver transcriptional activation to a particular genomic location in a guideRNA‐dependent manner^47^. In terms of gene repression, CRISPRinterference (CRISPRi) was described nearly half a decade ago, and refined by use of the Kruppel‐associated box (KRAB) domain, a repressor of transcription which can mediate efficient silencing in mammalian cells 48. KRAB domains are present in almost 400 human proteins, and exert their repressive capacity via recruitment of KRAB‐associated protein‐1 (Kap1) and heterochromatin protein‐1 (HP1) to mediate H3K9 trimethylation (Fig. 2a). These reagents can bypass the epigenetic constraints of gene expression within a cell and have been used for a variety of genomewide screens to efficiently silence either single or multiple genes or silence the influence of regulatory domains in the native genomic context 49, 50, 51, 52, 53, 54, 55, 56, 57, 58.

Guide RNA‐directed dCas9 fusion proteins have certain features that are advantageous over short interfering and short hairpin RNAs. A particular benefit is that, as regulators of the epigenome, the duration of efficacy is potentially longer than methodologies that target RNA degradation. When combined with the use of inducible promoters such as tetracycline‐induced transcriptional activation, precise control of modulation can be achieved.

More refined analysis, where the objective is to understand the functional consequence of delivery of specific methylation or acetylation modifying activities, are also beginning to be deployed. This approach has been conducted in fewer studies than have been undertaken using a more unbiased approach. Proteins that function as epigenetic regulators often have multiple modules mediating, among others, protein interaction and catalytic activity. Ideally, these modules might be isolated from one another to achieve specificity. Researchers have used the catalytic domain of p300 or an inactive mutant thereof to reconfigure the enhancer landscape and prove the involvement of histone acetylation at precise locations to alter gene expression potential 59, 60 (Fig. 2b). Similarly, fusion of histone or DNA methyl transferases to Cas9 has been used to prove the influence of specific DNA methylation events 61, 62, 63, 64, 65, 66.

Within the immune system only a few gene loci have been subjected to epigenome editing. Forkhead box protein 3 (FoxP3) regulatory domains were targeted with either dCas9.TET1 or dCas9.p300 catalytic domains in order to demethylate or deposit H3K27acetylation. While altered DNA methylation did not display a potent effect, histone acetylation promoted FoxP3 expression and induction of FoxP3 target genes 67, 68.

While fusion proteins of catalytic domains coupled to dCas9 can be used to deliver a particular histone or DNA modification artificially onto a chromatin template, they do not address how, in diseased cells, the altered chromatin landscape initiates, what transcription factors are differentially recruited and precisely which enzymes are the propagators of the altered landscape. These altered landscapes can, however, be interrogated to determine transcription factor binding‐site enrichment. To further this process of factor identification, epitope tagged dCas9 may also be used as an immunoprecipitation reagent, where guide RNAs provide the targeting capacity to specific regions of fragmented chromatin enabling unbiased analysis of the proteins that are recruited *in vivo* to those areas by mass spectrometry or immunological investigations 69 (Fig. 2c). This technology was used to identify factors binding to the IFN‐γ‐sensitive interferon regulatory factor 1 (IRF‐1) promoter 70. Furthermore, this system was exploited in order to identify non‐coding RNAs associating with telomeric regions.

## Outlook and summary

CRISPR.Cas9‐mediated epigenome editing is beginning to be employed to understand the epigenetic constraints upon gene expression, and in particular the impact of altered epigenomes in diseased cells. For such studies, however, numerous considerations arise. What is the cell type of interest? Is it possible to isolate those cells in health and disease? How many subjects should be studied? Are there distinct endotypes of disease that must be considered? Is there likely to by an impact of SNP variation on disease? Which histone modification should be studied? With the emergence of technologies permitting analysis of either single or few cells, this field of research is likely to be fertile, as the focus of analysis shifts from descriptive to analysis of functional consequence.

## Disclosures

The authors declare no competing financial interests.

## References

[1] Lander ES , Linton LM , Birren B *et al* Initial sequencing and analysis of the human genome. Nature 2001; 409:860–921. 11237011 10.1038/35057062

[2] Bernstein BE , Stamatoyannopoulos JA , Costello JF *et al* The NIH roadmap epigenomics mapping consortium. Nat Biotechnol 2010; 28:1045–8. 20944595 10.1038/nbt1010-1045 PMC3607281

[3] ENCODE Project Consortium . An integrated encyclopedia of DNA elements in the human genome. Nature 2012; 489:57–74. 22955616 10.1038/nature11247 PMC3439153

[4] Villani AC , Satija R , Reynolds G *et al* Single‐cell RNA‐seq reveals new types of human blood dendritic cells, monocytes, and progenitors. Science 2017; 356. doi: https://doi.org/10.13140/RG.2.2.25417.13922. 10.1126/science.aah4573 PMC5775029 28428369

[5] Regev A , Teichmann SA , Lander ES *et al* Human cell atlas meeting participants. The human cell atlas. eLife 2017; doi: 10.7554/eLife.27041.

[6] Jiang X , Clark RA , Liu L , Wagers AJ , Fuhlbrigge RC , Kupper TS . Skin infection generates non‐migratory memory CD8+ TRM cells providing global skin immunity. Nature 2012;483:227–31. 22388819 10.1038/nature10851 PMC3437663

[7] van der Maaten LJP , Hinton GE . Visualizing high‐dimensional data using t‐SNE. J Mach Learn Res 2008; 9:2579–605.

[8] Kelsey G , Stegle O , Reik W . Single‐cell epigenomics: recording the past and predicting the future. Science 2017; 358:69–75. 28983045 10.1126/science.aan6826

[9] Skene PJ , Henikoff S . An efficient targeted nuclease strategy for high‐resolution mapping of DNA binding sites. eLife 2017; 16:6. 10.7554/eLife.21856 PMC5310842 28079019

[10] Schmid M , Durussel T , Laemmli UK . ChIC and ChEC; genomic mapping of chromatin proteins. Mol Cell 2004;16:147–57. 15469830 10.1016/j.molcel.2004.09.007

[11] Lieberman‐Aiden E , van Berkum NL , Williams L *et al* Comprehensive mapping of long‐range interactions reveals folding principles of the human genome. Science 2009;326:289–93. 19815776 10.1126/science.1181369 PMC2858594

[12] Dixon JR , Selvaraj S , Yue F *et al* Topological domains in mammalian genomes identified by analysis of chromatin interactions. Nature 2012; 485:376–80. 22495300 10.1038/nature11082 PMC3356448

[13] Kouzarides T . Chromatin modifications and their function. Cell 2007; 128:693–705. 17320507 10.1016/j.cell.2007.02.005

[14] Barski A , Cuddapah S , Cui K *et al* High‐resolution profiling of histone methylations in the human genome. Cell 2007; 129:823–37. 17512414 10.1016/j.cell.2007.05.009

[15] Creyghton MP , Cheng AW , Welstead GG *et al* Histone H3K27ac separates active from poised enhancers and predicts developmental state. Proc Natl Acad Sci USA 2010; 107:21931–6. 21106759 10.1073/pnas.1016071107 PMC3003124

[16] Heintzman ND , Hon GC , Hawkins RD *et al* Histone modifications at human enhancers reflect global cell‐type‐specific gene expression. Nature 2009; 459:108–12. 19295514 10.1038/nature07829 PMC2910248

[17] Rada‐Iglesias A , Bajpai R , Swigut T , Brugmann SA , Flynn RA , Wysocka J . A unique chromatin signature uncovers early developmental enhancers in humans. Nature 2011;470:279–83. 21160473 10.1038/nature09692 PMC4445674

[18] Kolasinska‐Zwierz P , Down T , Latorre I , Liu T , Liu XS , Ahringer J . Differential chromatin marking of introns and expressed exons by H3K36me3. Nat Genet 2009; 41:376–81. 19182803 10.1038/ng.322 PMC2648722

[19] Struhl K . Histone acetylation and transcriptional regulatory mechanisms. Genes Dev 1998; 12:599–606. 9499396 10.1101/gad.12.5.599

[20] Zhou W , Zhu P , Wang J *et al* Histone H2A monoubiquitination represses transcription by inhibiting RNA polymerase II transcriptional elongation. Mol Cell 2008; 29:69–80. 18206970 10.1016/j.molcel.2007.11.002 PMC2327256

[21] Grant PA , Schieltz D , Pray‐Grant MG *et al* A subset of TAF(II)s are integral components of the SAGA complex required for nucleosome acetylation and transcriptional stimulation. Cell 1998; 94:45–53. 9674426 10.1016/s0092-8674(00)81220-9

[22] Pirrotta V . Polycombing the genome: PcG, trxG, and chromatin silencing. Cell 1998; 93:333–6. 9590168 10.1016/s0092-8674(00)81162-9

[23] Kwon H , Imbalzano AN , Khavari PA , Kingston RE , Green MR . Nucleosome disruption and enhancement of activator binding by a human SW1/SNF complex. Nature 1994; 370:477–81. 8047169 10.1038/370477a0

[24] Doi A , Park IH , Wen B *et al* Differential methylation of tissue‐ and cancer‐specific CpG island shores distinguishes human induced pluripotent stem cells, embryonic stem cells and fibroblasts. Nat Genet 2009; 41:1350‐3. 19881528 10.1038/ng.471 PMC2958040

[25] Zhang X , Yang Z , Khan SI *et al* Structural basis for the product specificity of histone lysine methyltransferases. Nat Genet 2009; 12:177–185. 10.1016/s1097-2765(03)00224-7 PMC2713655 12887903

[26] Bedford MT , Clarke SG . Protein arginine methylation in mammals: who, what, and why. Mol Cell 2009; 33:1–13. 19150423 10.1016/j.molcel.2008.12.013 PMC3372459

[27] Shi Y , Lan F , Matson C *et al* Histone demethylation mediated by the nuclear amine oxidase homolog LSD1. Cell 2004; 119:941–53. 15620353 10.1016/j.cell.2004.12.012

[28] Mosammaparast N , Shi Y . Reversal of histone methylation: biochemical and molecular mechanisms of histone demethylases. Annu Rev Biochem 2010; 79:155–79. 20373914 10.1146/annurev.biochem.78.070907.103946

[29] Neuwald AF , Landsman D . GCN5‐related histone N‐acetyltransferases belong to a diverse superfamily that includes the yeast SPT10 protein. Trends Biochem Sci 1997; 22:154–5. 9175471 10.1016/s0968-0004(97)01034-7

[30] Utley RT , Côté J . The MYST family of histone acetyltransferases. Curr Top Microbiol Immunol 2003; 274:203–36. 12596909 10.1007/978-3-642-55747-7_8

[31] Bannister AJ , Kouzarides T . The CBP co‐activator is a histone acetyltransferase. Nature 1996; 384:641–3. 8967953 10.1038/384641a0

[32] Grozinger CM , Hassig CA , Schreiber SL . Three proteins define a class of human histone deacetylases related to yeast Hda1p. Proc Natl Acad Sci USA 1999; 96:4868–73. 10220385 10.1073/pnas.96.9.4868 PMC21783

[33] Fischle W , Kiermer V , Dequiedt F , Verdin E . The emerging role of class II histone deacetylases. Biochem Cell Biol 2001; 79:337–48. 11467747

[34] Vaquero A , Sternglanz R , Reinberg D . NAD+‐dependent deacetylation of H4 lysine 16 by class III HDACs. Oncogene 2007; 26:5505–20. 17694090 10.1038/sj.onc.1210617

[35] Okano M , Bell DW , Haber DA , Li E . DNA methyltransferases Dnmt3a and Dnmt3b are essential for de novo methylation and mammalian development. Cell 1999; 99:247–57. 10555141 10.1016/s0092-8674(00)81656-6

[36] Tahiliani M , Koh KP , Shen Y *et al* Conversion of 5‐methylcytosine to 5‐hydroxymethylcytosine in mammalian DNA by MLL partner TET1. Science 2009; 324:930–935. 19372391 10.1126/science.1170116 PMC2715015

[37] Ficz G , Branco MR , Seisenberger S *et al* Dynamic regulation of 5‐hydroxymethylcytosine in mouse ES cells and during differentiation. Nature 2011; 473:398–402. 21460836 10.1038/nature10008

[38] Wossidlo M , Nakamura T , Lepikhov K *et al* 5‐Hydroxymethylcytosine in the mammalian zygote is linked with epigenetic reprogramming. Nat Commun 2011; 2011:1–8. 10.1038/ncomms1240 21407207

[39] Xu Y , Zhang S , Lin S *et al* WERAM: a database of writers, erasers and readers of histone acetylation and methylation in eukaryotes. Nucleic Acids Res 2017; 45:D264–70. 27789692 10.1093/nar/gkw1011 PMC5210520

[40] Ballestar E , Wolffe AP . Methyl‐CpG‐binding proteins. Targeting specific gene repression. Eur J Biochem 2001; 268:1–6. 11121095 10.1046/j.1432-1327.2001.01869.x

[41] Blackledge NP , Zhou JC , Tolstorukov MY , Farcas AM , Park PJ , Klose RJ . CpG islands recruit a histone H3 lysine 36 demethylase. Mol Cell 2010; 38:179–90. 20417597 10.1016/j.molcel.2010.04.009 PMC3098377

[42] Prinjha RK , Witherington J , Lee K . Place your BETs: the therapeutic potential of bromodomains. Trends Pharmacol Sci 2012; 33:146–53. 22277300 10.1016/j.tips.2011.12.002

[43] Dawson MA , Prinjha RK , Dittmann A *et al* Inhibition of BET recruitment to chromatin as an effective treatment for MLL‐fusion leukaemia. Nature 2011; 478:529–33. 21964340 10.1038/nature10509 PMC3679520

[44] Pabo CO , Peisach E , Grant RA . Design and selection of novel Cys2His2 zinc finger proteins. Annu Rev Biochem 2001; 70:313–40. 11395410 10.1146/annurev.biochem.70.1.313

[45] Bogdanove AJ , Voytas DF . TAL effectors: customizable proteins for DNA targeting. Science 2011; 333:1843–6. 21960622 10.1126/science.1204094

[46] Charpentier E , Doudna JA . Biotechnology: rewriting a genome. Nature 2013; 495:50–1. 23467164 10.1038/495050a

[47] Balboa D , Weltner J , Eurola S , Trokovic R , Wartiovaara K , Otonkoski T . Conditionally stabilized dCas9 activator for controlling gene expression in human cell reprogramming and differentiation. Stem Cell Rep 2015; 5:448–59. 10.1016/j.stemcr.2015.08.001 PMC4618656 26352799

[48] Groner AC , Meylan S , Ciuffi A *et al* KRAB‐zinc finger proteins and KAP1 can mediate long‐range transcriptional repression through heterochromatin spreading. PLOS Genet 2010; 6:1–14. 10.1371/journal.pgen.1000869 PMC2832679 20221260

[49] Parsi KM , Hennessy E , Kearns N , Maehr R . Using an inducible CRISPR‐dCas9‐KRAB effector system to dissect transcriptional regulation in human embryonic stem cells. Methods Mol Biol 2017; 1507:221–33. 27832543 10.1007/978-1-4939-6518-2_16

[50] Amabile A , Migliara A , Capasso P *et al* Inheritable silencing of endogenous genes by hit‐and‐run targeted epigenetic editing. Cell 2016; 167:219–32. 27662090 10.1016/j.cell.2016.09.006 PMC5039111

[51] Ecco G , Imbeault M , Trono D . KRAB zinc finger proteins. Development 2017; 144:2719–29. 28765213 10.1242/dev.132605 PMC7117961

[52] Korkmaz G , Lopes R , Ugalde AP *et al* Functional genetic screens for enhancer elements in the human genome using CRISPR‐ Cas9. Nat Biotechnol 2016; 34:192–98. 26751173 10.1038/nbt.3450

[53] Rajagopal N , Srinivasan S , Kooshesh K *et al* High‐throughput mapping of regulatory DNA. Nat Biotechnol 2016; 34:167–74. 26807528 10.1038/nbt.3468 PMC5108523

[54] Diao Y , Li B , Meng Z *et al* A new class of temporarily phenotypic enhancers identified by CRISPR/Cas9‐mediated genetic screening. Genome Res 2016; 26:397–405. 26813977 10.1101/gr.197152.115 PMC4772021

[55] Sanjana NE , Wright J , Zheng K *et al* High‐resolution interrogation of functional elements in the noncoding genome. Science 2016; 353:1545–9. 27708104 10.1126/science.aaf7613 PMC5144102

[56] Vierstra J , Reik A , Chang KH *et al* Functional footprinting of regulatory DNA. Nat Methods 2015; 12:927–30. 26322838 10.1038/nmeth.3554 PMC5381659

[57] Fulco CP , Munschauer M , Anyoha R *et al* Systematic mapping of functional enhancer‐promoter connections with CRISPR interference. Science 2016; 354:769–73. 27708057 10.1126/science.aag2445 PMC5438575

[58] Gilbert LA , Horlbeck MA , Adamson B *et al* Genome‐scale crispr‐mediated control of gene repression and activation. Cell 2014; 159:647–61. 25307932 10.1016/j.cell.2014.09.029 PMC4253859

[59] Hilton IB , D’Ippolito AM , Vockley CM *et al* Epigenome editing by a CRISPR‐Cas9‐based acetyltransferase activates genes from promoters and enhancers. Nat Biotechnol 2015; 33:510–17. 25849900 10.1038/nbt.3199 PMC4430400

[60] Thakore PI , D’Ippolito AM , Song L *et al* Highly specific epigenome editing by CRISPR‐Cas9 repressors for silencing of distal regulatory elements. Nat Methods 2015; 12:1143–9. 26501517 10.1038/nmeth.3630 PMC4666778

[61] Liu XS , Wu H , Ji X *et al* Editing DNA methylation in the mammalian genome. Cell 2016; 167:233–47. 27662091 10.1016/j.cell.2016.08.056 PMC5062609

[62] Vojta A , Dobrinić P , Tadić V *et al* Repurposing the CRISPR‐Cas9 system for targeted DNA methylation. Nucleic Acids Res 2016; 44:5615–28. 26969735 10.1093/nar/gkw159 PMC4937303

[63] McDonald JI , Celik H , Rois LE *et al* Reprogrammable CRISPR/Cas9‐based system for inducing site‐specific DNA methylation. Biol Open 2016; 5:866–74. 27170255 10.1242/bio.019067 PMC4920199

[64] Xu X , Tao Y , Gao X *et al* A CRISPR‐based approach for targeted DNA demethylation. Cell Discov 2016; 2. doi: 10.1038/celldisc.2016.9. PMC4853773 27462456

[65] Stepper P , Kungulovski G , Jurkowska RZ *et al* Efficient targeted DNA methylation with chimeric dCas9‐Dnmt3a‐Dnmt3L methyltransferase. Nucleic Acids Res 2017; 45:1703–13. 27899645 10.1093/nar/gkw1112 PMC5389507

[66] Huang YH , Su J , Lei Y *et al* DNA epigenome editing using CRISPR‐Cas SunTag‐directed DNMT3A. Genome Biol 2017; 18:176. 28923089 10.1186/s13059-017-1306-z PMC5604343

[67] Okada M , Kanamori M , Someya K , Nakatsukasa H , Yoshimura A . Stabilization of Foxp3 expression by CRISPR‐dCas9‐based epigenome editing in mouse primary T cells. Epigenet Chromatin 2017; 10:24. 10.1186/s13072-017-0129-1 PMC5422987 28503202

[68] Someya K , Nakatsukasa H , Ito M *et al* Improvement of Foxp3 stability through CNS2 demethylation by TET enzyme induction and activation. Int Immunol 2017; 29:365–75. 29048538 10.1093/intimm/dxx049 PMC5890887

[69] Fujita T , Fujii H . Efficient isolation of specific genomic regions and identification of associated proteins by engineered DNA‐binding molecule‐mediated chromatin immunoprecipitation (enChIP) using CRISPR. Biochem Biophys Res Commun 2013; 439:132–6. 23942116 10.1016/j.bbrc.2013.08.013

[70] Fujita T , Fujii H . Identification of proteins associated with an IFNγ‐responsive promoter by a retroviral expression system for enChIP using CRISPR. PLOS ONE 2014; 9:e103084. 25051498 10.1371/journal.pone.0103084 PMC4106880

